# Molecular epidemiology of anaplasmosis in small ruminants along a human-livestock-wildlife interface in Uganda

**DOI:** 10.1016/j.heliyon.2020.e05688

**Published:** 2020-12-31

**Authors:** Keneth Iceland Kasozi, Susan Christina Welburn, Gaber El-Saber Batiha, Najat Marraiki, David Paul Nalumenya, Monica Namayanja, Kevin Matama, Kelly Katenta Zalwango, Wycliff Matovu, Gerald Zirintunda, Justine Ekou, Stellamaris Kembabazi, Claire Mack Mugasa, Annah Kitibwa, Dickson Stuart Tayebwa, Simon Peter Musinguzi, Michael Mahero, Ibrahim Ssengendo, Anne Nanteza, Enock Matovu, Ewan Thomas MacLeod

**Affiliations:** a Infection Medicine, Deanery of Biomedical Sciences, College of Medicine and Veterinary Medicine, The University of Edinburgh, 1 George Square, Edinburgh EH8 9JZ, United Kingdom; b Department of Animal Production and Management, Faculty of Agriculture and Agricultural Sciences, Busitema University Arapai Campus, Box 203 Soroti, Uganda; c School of Medicine, Kabale University, Box 317 Kabale, Uganda; d Zhejiang University-University of Edinburgh Institute, Zhejiang University School of Medicine, International Campus, 718 East Haizhou Road, Haining 314400, China; e Department of Pharmacology and Therapeutics, Faculty of Veterinary Medicine, Damanhour University, Damanhour 22511, AlBeheira, Egypt; f Department of Botany and Microbiology, College of Science, King Saud University, Riyadh 11451, Saudi Arabia; g College of Veterinary Medicine Animal Resources and Biosecurity, Makerere University, Box 7062 Kampala, Uganda; h Kampala International University Western Campus, Box 71 Bushenyi, Uganda; i Faculty of Agriculture and Environmental Sciences, Kabale University, Box 315 Kabale, Uganda; j Department of Veterinary Population Medicine, College of Veterinary Medicine, University of Minnesota, USA

**Keywords:** Tick-borne diseases, *Anaplasma ovis*, Parasites, Small ruminants, Goats, Uganda, Africa, Ticks, Tick resistance, Tick vaccines, Entomology, Ecosystem services, Protozoa, Polymerase chain reaction, Epidemiology, Veterinary medicine, Animal behavior, Animal breeding, Ruminant, Agriculture

## Abstract

**Background:**

Information as regards the epidemiology of the *Anaplasmataceae* in small ruminants in several low- and middle-income countries is scarce.

**Methods:**

In this study a total of 712 DNA samples collected from small ruminants were analyzed for *Anaplasmataceae* and *Anaplasma ovis* using the *16S rRNA* and *MSP4* genes respectively. Infection risk was assessed by location, sex and age of the animals and qGIS® was used to construct spatial maps.

**Results:**

The prevalence of *Anaplasmataceae* spp was 89.1% (95% CI: 77.5–95.9) and 79.1% (95% CI: 75.9–82.1) in ovines and caprines respectively (RR = 1.1, 95% CI: 1.0–1.3); higher than those previously reported in other eastern African countries. The prevalence of *A. ovis* was 26.1% and 25.4% for both ovines and caprines respectively with ovines showing significantly higher levels of infection than caprines (P < 0.05). The risk of *Anaplasma ovis* infections was not affected by age (OR = 1.2, 95% CI: 0.9–1.7) or sex (OR = 1.1, 95% CI: 0.6–2.0). Small ruminants located at the forest edge (<0.3 km) showed higher *A. ovis* prevalence than those found inland with infections present in the midland regions associated with increased agricultural activity.

**Conclusion:**

*Anaplasma ovis* remains a major challenge for small ruminant husbandry in Uganda and infections are under-reported. Policy efforts to prioritize management of *Anaplasmataceae* for small ruminant health would promote livestock productivity in vulnerable communities, improving livelihoods and ecosystem health.

## 1 Introduction

Across Africa and Asia, small ruminants often maintained in mixed farms, are a major source of animal protein and raw materials for the fragile food industry (Devendra, 1994). In communities where poverty is endemic, small ruminants contribute essential revenue (Peacock, 2005), assets (Inn, 2003), and sources of food security (Herrero et al., 2013; Lalljee et al., 2019). Small ruminants are an important pathway towards the realization of the 2030 Sustainable Development Goal (SDG) targets (Food and Agriculture Organization, 2018) in vulnerable communities, contributing to: zero poverty (goal 1), good health and well-being (goal 2); decent work and economic growth (goal 8). Small ruminants are considered key to fighting poverty in Uganda (Akejo and Otto, 2017; Inn, 2003) but the increasing burden of hemoprotozoan infections threatens their productivity and community livelihoods due to the risk of zoonotic disease transmission (Kasozi et al., 2019). Most small ruminants that are kept are indigenous, having advantages over exotics in terms of climate resilience and being considered low maintenance (Monau et al., 2020). Tick-borne infections are however a major productivity challenge (Bilgic et al., 2017).

Several studies have been conducted on the epidemiology of tick-borne infections in cattle due to their high economic value (Bardosh et al., 2013; Vudriko et al., 2016; Weny et al., 2017). In Uganda, major Ixoid tick species identified include *Rhipicephalus* species (especially *Rhipicephalus appendiculatus*), *Amblyomma* species (especially *Amblyomma variegatum*) and *Hyalomma* species (*Hyalomma rufipes* and *Hyalomma truncatum*) continue to be common in bovines (Balinandi et al., 2020) and these species continue to be present nationwide (Muhanguzi et al., 2020).

A polymerase chain reaction (PCR) based study (Ikwap et al., 2010) identified new and unknown *Anaplasma* genotypes in bovines from Uganda. Rather less emphasis has been placed on small ruminants. *A ovis* is responsible for the majority of anaplasmosis cases in small ruminants (Bilgic et al., 2017; Torina et al., 2010) and there is a need to prioritize studies in eastern Africa to affect improvements in small ruminant health. Since many countries in eastern Africa rely on small ruminants for food and income (Herrero et al., 2013; Inn, 2003; Lalljee et al., 2019; Peacock, 2005), attainment of the SDGs in the region will require national governments to revise their disease control policies.

Recent surveys show increasing evidence of *Anaplasma* spp in small ruminants. The prevalence of *Anaplasma* spp was found to be 40.8% in ovines from central and western Kenya by PCR (Ringo et al., 2019). In northeastern Uganda, a prevalence of 19.5% for *Anaplasma* spp in small ruminants was reported by microscopy (Lolli et al., 2016). In Sudan, a prevalence of 60.1% for *A. ovis* with prevalence of infection being higher in ovines than caprines following PCR analysis (Lee et al., 2018). In Tunisia, *Anaplasma* spp. and *A. ovis* prevalence were 95.0% and 93.8% in ovines and 69.6% and 65.3% in caprines, respectively while no *A. phagocytophilum* was detected by PCR (Said et al., 2015). In Senegal, prevalence of *Anaplasmataceae* infection was 41.1% and in these, *Anaplasma ovis* 55.9% in ovines, *A. marginale* and *A. centrale* in 19.4% and 8.1% in bovines and putative new species of *Anaplasmataceae* were found by PCR (Dahmani et al., 2019). A study from Turkey, Iraq, Sudan, and Portugal on *Anaplasma ovis* established a prevalence of 31.4%, 66.6% 41.6% and 82.5%, respectively, indicating that the high prevalence of *A. ovis* in these countries, calling for action to stop the neglect of these bacterial infections in small ruminants (Renneker et al., 2013). This showed that molecular studies estimating the prevalence of anaplasmosis in small ruminants of Uganda are scarce, this is despite mounting epidemiological evidence of their importance as maintenance hosts for *Anaplasmatacea* in Ugandan livestock communities.

Molecular diagnosis of *Anaplasma* bacteria is by detection of the 16S rRNA gene (Ringo et al., 2019; Shi et al., 2020; Yang et al., 2016). This forms the basis of phylogenetic analysis in the *Anaplasmataceae* (Seo et al., 2018) for insight into genotypic variation and diversity. In Xinjiang, northwest China, a prevalence of 17.6% for *A. phagocytophilum,* 4.8% for *A. bovis* and 40.5% for *A. ovis* shows the importance of these bacteria in small ruminant health (Yang et al., 2015). For *A. ovis* diagnosis, the *MSP4* gene is the a major target (Ali et al., 2017).

This study aimed to assess the prevalence of *Anaplasmataceae* in small ruminants of Uganda Prior to this study, examination of *A. ovis* in small ruminants had never been conducted in Uganda.

## 2 Material and methods

### 2.1 Study design

**Figure 1:**
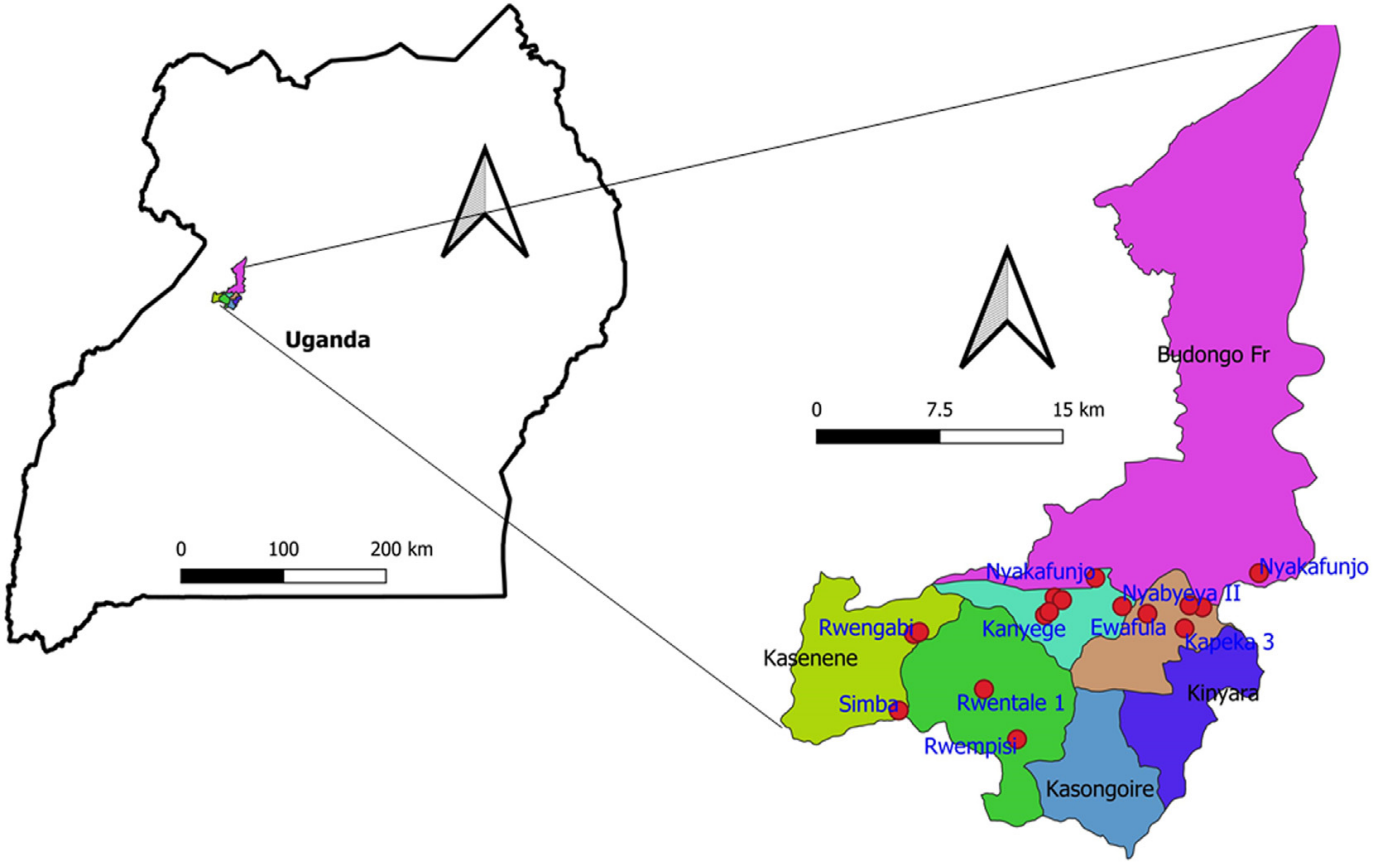
**Description of the study area.** Samples were collected in georeferenced villages within Budongo subcounty of Masindi district (Uganda). Animals were located within homesteads which were close to each other as observed from the diffuse village names especially in Nyabyeya parish. This proximity allows for communal grazing to maximize utilization of the minimal pasture grounds at the forest edge, thus creating an environment with fosters human-wildlife conflicts.

This cross sectional study was conducted on 712 DNA samples obtained from small ruminants at the fringes of Budongo Conservation Forest in Masindi district, Western Uganda (Figure 1). Small ruminants included were only ovines and caprines sampled since these were the prevalent (90%) animal species in the community using records from the veterinary officer at the local subcounty office. Sampled villages were chosen randomly using a random assortment algorithm in MS Excel 2019 in consultation with the local community leaders (Kasozi et al., 2019).

### 2.2 Blood collection and DNA extraction

Blood samples were aseptically collected from the jugular vein of the survey animals, placed in a redtop vacutainer and stored at 4 °C. DNA was extracted using Himedia® HiPura™ blood genomic DNA Mini-prep purification spin kit with minor modifications (Jaswal et al., 2014). In an Eppendorf, 300 μl of blood was added to 900 μl of RBC lysis solution, incubated for 3 min at room temperature. This was then centrifuged at max speed (16,000 relative centrifugal force) for 1 min and the supernatant was discarded. The pellet this was resuspended in the residual fluid. To the tube containing the residual fluid, 150 μl of cell lysis solution was added, followed by 50 μl of protein precipitation solution. This was vortexed vigorously for 20 s, spun for 2 min at max speed. In a clean Eppendorf tube with labels transferred, 150 μl of isopropanol (100%), and the supernatant after the spin was added to the isopropanol. This was then mixed gently by inverting the tube 50 times, then centrifuged at max speed for 2 min. The supernatant was then discarded, leaving only the pellet. Finally, 150 μl of ethanol was added to wash the pellet and this was centrifuged at max speed for 2 min. The supernatant was then discarded and the tube was drained and left in the PCR hood to dry. To the dried tube, 50 μl of DNA hydration solution was added and the mixture was incubated at 65 °C for 10 min, mixed gently by pipetting and stored at 4 °C for routine use and -20 °C for future reference.

### 2.3 Molecular identification of *Anaplasmataceae* in small ruminants

PCR targeting the *Anaplasmataceae* 16S rRNA gene was used to detect *Anaplasma* spp (Ringo et al., 2019) with minor modifications. A 10 μL reaction containing 1x My Taq mix (Bioline®), about 1 μg of genomic DNA 0.2 μM each of the forward and reverse primers. Primer nucleotide sequences used were: forward primers, 5′GGTTTAATTCGATGCAACGCGA-3′, and reverse primers 5′CGTATTCACCGTGGCATG 3’ (Bekker et al., 2002; Ringo et al., 2019). *Anaplasma centrale* DNA was used as a positive control (Ikwap et al., 2010). After an initial denaturation step at 95 °C for 5 min, the PCR amplifications were performed for 45 cycles, each contained a denaturation step at 95 °C for 30 s, an annealing step at 74 °C for 30 s and an extension step at 72 °C for 45s. PCR products were then resolved by gel electrophoresis in a 1.5% agarose gel, stained with ethidium bromide, and visualized under a UV transilluminator, the PCR amplicons of 430 bp were considered as positive for *Anaplasmataceae* after visualization alongside the 1000 bp DNA ladder.

### 2.4 Molecular identification of *Anaplasma ovis* in small ruminants

The *MSP4* gene of *Anaplasma ovis* with GenBank no. HQ456350.1 was amplified using forward primer 5TGAAGGGAGCGGGGTCATGGG3 with reverse primer 5′ GGTAATTGCAGCCAGGGACTCT 3′ were used (Ali et al., 2017). The PCR mix consisted of the primers each at a final concentration of 0.2μM, 1x My Taq mix (Bioline®) and about 1 μg of genomic DNA. The PCR was done following a cycling program that consisted of an initial denaturation step of 5 min at 95 °C, each cycle consisting of a denaturing step of 30 sat 95 °C, an annealing temperature for 30 s at 60 °C and an extension step of 45 s at 72 °C for 35 cycles. PCR was completed with the additional extension step for 10 min at 72 °C. The positive control for *A. ovis* from the study was used. PCR products were run on a 1.5% agarose gel and visualized under UV transilluminator to detect a PCR product of 347 bp.

### 2.5 Ecological distribution of *Anaplasmataceae* in the study area

Georeferenced village survey points were imported into quantitative geographical system (qGIS®) open-source software. Using an image acquired from the United States Geographical Surveys (USGS) Aster global Dem Satellite image number ASTGM2_N01E031_dem land elevation analysis was done and vegetation cover analysis was conducted using an image acquired from USGS Sentinel-2 satellite, image number L1C_T36NUG_A013088_20171224T091914. The satellite image files were modified to show 4 levels of classifications, band 1 (Red) with rendering type of single band pseudocolor linear using settings in qGIS®. Unit 1 was lake vegetation (purple), 46.3 units for budongo forest vegetation (blue), 91.7 units for sugarcane and community agricultural cultivation (green) and 137 units for bare soil (yellow). Digital land elevation was divided into 4 categories i.e. 592 units (color deep blue) for lake level, 1055 units (light blue) low land, 1095 units (brown color) midlands and 1440 and above (red color) on highlands. Vegetation land cover satellite images were divided into 3 categories i.e. 1-unit (purple color) for Budongo forest, 69 units (blue color) community crops and sugarcane plantation and 137 units (yellow color) for soil with no vegetation were used to define risk areas for infection in the study area and presented on maps.

### 2.6 Ethical approval

Ethical approval from the Ethical Review Board at the University of Edinburgh and clearance from the Uganda National Council of Science and Technology with reference numbers OS5-17 and A570 respectively were acquired.

### 2.7 Statistical analysis

Data were recorded in Microsoft Excel and descriptive statistics were conducted using WinPepi® to determine the disease risks and their 95% confidence interval, for infections in small ruminants. Significance was reported when the 95% confidence interval didn't contain the null value.

## 3 Results

### 3.1 Prevalence of *Anaplasmataceae* in small ruminants of Uganda

The study showed an *Anaplasmataceae* prevalence of 79.1% (95% CI: 75.9–82.1) and 89.1% (95% CI: 77.5–95.9) in caprines and ovines respectively. Infections in small ruminants due to *Anaplasmataceae* were significantly higher in ovines than caprines (RR = 1.1, 95% CI: 1.0–1.3). Prevalence of *Anaplasma ovis* was 25.4% (95% CI: 22.2–28.8) and 26.1%, (95% CI: 15.6–40.3) for both caprines and ovines respectively and there were no significant differences between infections in caprines and ovines (RR = 1.0, 95% CI: 0.6–1.7) as shown in Table 1.

**Table 1:** Prevalence of Anaplasmataceae in small ruminants of Uganda.

	Species	Frequency (prevalence; 95% CI)	Risk of infections		
			RR (95% CI)	OR (95% CI)	aR (95% CI)
*Anaplasmataceae*	Ovines (n = 46)	41 (89.1; 77.5–95.9)	1.1 (1.0–1.3)	2.2 (0.9–6.3)	10 (-0.7–20.7)
	Caprines (n = 666)	527 (79.1; 75.9–82.1)			
	Total (n = 712)	568 (79.8; 76.7–82.6)			
*Anaplasma ovis*	Ovines (n = 46)	12 (26.1; 15.0–40.1)	1.0 (0.6–1.7)	1.0 (0.5–2.0)	0.7 (-13.6–15.0)
	Caprines (n = 666)	169 (25.4; 22.2–28.8)			
	Total (n = 712)	181 (25.4; 22.3–28.7)			

**KEY:** RR = relative risk, OR = odds ratios and aR = attributable risk.

### 3.2 Major risk factors for anaplasmosis in small ruminants of Uganda

*Anaplasmataceae* infections in adults and juveniles were not observed to be significantly different (RR = 1.0, 95% CI: 1.0–1.1) and similar observations were found amongst males and females and location. No difference (OR = 1.2, 95% CI: 0.9–1.7) was found in the prevalence of *A. ovis* between juveniles and adults. There was also no difference (OR = 1.1, 95% CI: 0.6–2.0) between sex of male prevalence (27.8%; 95% CI: 18.8–39.1) and female prevalence (25.2%, 95% CI: 22.0–28.7) of small ruminants (Table 2). *Anaplasmataceae* prevalence was highest in communities <0.3 km from the forest edge than those far away i.e. 82.6% vs 79.1%. *A. ovis* prevalence was higher in communities closer to the forest edge than those more than 0.3 km from the forest edge (24.9%) although no significant differences were found (OR = 1.2, 95% CI: 0.7–1.8).

**Table 2:** Factors precipitating anaplasmosis infections in small ruminants of Uganda.

Parameter	Variable	Frequency (%) in small ruminants			Risk of infections			P-value
		Positive	Negative	Total	RR (95% CI)	OR (95% CI)	aR (95% CI)	
*Anaplasmataceae*								
Age	Adult	349 (80.4)	85 (19.6)	434 (100)	1.0 (1.0–1.1)	1.1 (0.8–1.6)	1.6 (-4.7–8.0)	
	Juvenile	219 (78.8)	59 (21.2)	278 (100)				
Sex	Male	59 (81.9)	13 (18.1)	72 (100)	1.0 (0.9–1.2)	1.2 (0.6–2.3)	2.4 (-7.8–12.6)	
	Female	509 (79.5)	131 (20.5)	640 (100)				
Location (parish)	Kabango	193 (75.7)	62 (24.3)	255 (100)	3.1	1	51.4	1
	Kasenene	90 (83.3)	18 (16.7)	108 (100)	5.0	1.6	66.7	2.7 (0.6)
	Nyabyeya	186 (81.2)	43 (18.8)	229 (100)	4.3	1.4	62.5	2.2 (0.8)
	Nyantonzi	99 (82.5)	21 (17.5)	120 (100)	4.7	1.5	65.0	2.3 (0.8)
Encroachment on forest edge	≤0.3 km	114 (82.6)	24 (17.4)	138 (100)	1.0 (1.0–1.1)	1.3 (0.8–2.1)	3.5 (-4.1–11.1)	
	>0.3 km	454 (79.1)	120 (20.9)	574 (100)				
*Anaplasma ovis*								
Age	Juvenile	77 (27.7)	201 (72.3)	278 (100)	1.2 (0.9–1.5)	1.2 (0.9–1.7)	3.7 (-3.2–10.6)	
	Adult	104 (24.0)	330 (76.0)	434 (100)				
Sex	Male	20 (27.8)	52 (72.2)	72 (100)	1.1 (0.7–1.6)	1.1 (0.6–2.0)	2.6 (-9.0–14.3)	
	Female	161 (25.2)	479 (74.8)	640 (100)				
Location (Parish)	Kabango	60 (23.5)	195 (76.5)	255 (100)	0.3	1	-52.9	1
	Kasenene	27 (25.0)	81 (75.0)	108 (100)	0.3	1.1	-50.0	0.9 (1.0)
	Nyabyeya	73 (31.9)	156 (68.1)	229 (100)	0.5	1.5	-36.2	4.2 (0.1)
	Nyantonzi	21 (17.5)	99 (82.5)	120 (100)	0.2	0.7	-65.0	1.8 (0.5)
Encroachment on forest edge	≤0.3 km	38 (27.5)	100 (72.5)	138 (100)	1.1 (0.8–1.5)	1.2 (0.7–1.8)	2.6 (-6.1–11.3)	0.5 (0.5)
	>0.3 km	143 (24.9)	431 (75.1)	574 (100)				

**KEY:** RR = relative risk, OR = odds ratios and aR = attributable risk.

### 3.3 *Anaplasmataceae* prevalence in the surveyed villages amongst small ruminants

The prevalence in small ruminants attributable to *Anaplasmataceae* was in the order of Rwempisi > Kapeka 3 > Rwentale 1 > Rwengabi > Simba > Ewafula > Nyabyeya 2 > Maraam > Nyakafunjo > Kapeka 1 > Kapeka 2 > Kanyege > Kalongo while *Anaplasma ovis* prevalence was in the order of Kalongo > Kanyege > Nyabyeya 2 > Maraam > Ewafula > Nyakafunjo > Simba > Rwengabi > Kapeka 2 > Rwentale 1 > Kapeka 1 > Kapeka 3 > Rwempisi and significantly higher differences in *Anaplasmataceae* than *Anaplasma ovis* prevalence were found (P < 0.05).

The prevalence of *Anaplasma ovis* ranked in this order Kalongo > Kanyege > Nyabyeya > Maraam > Ewafula > Nyakafunjo > Simba > Rwengabi > Kapeka 2 > Rwentale 1 > Kapeka 1 > Kapeka 3 > Rwempisi (Table 3). The odds ratio following infection with other *Anaplasma* parasites other than A. ovis was 28 times in Kalongo, 22 times in Kapeka 3, 21 times in Rwentale 1, 15 times in Rwengabi and Simba respectively.

**Table 3:** Village prevalence of *Anaplasmataceae* and infection risk estimates in western Uganda.

Village (n), distance in km from forest	*Anaplasma* spp. prevalence			*Anaplasma ovis* prevalence			Risk estimates		
	Freq (%)	95% CI	Ranking	Freq (%)	95% CI	Ranking	aR (95% CI)	RR (95% CI)	OR (95% CI)
Ewafula (68), 1.0	58 (85.3)	75.4–92.3	6	19 (27.9)	18.3–39.5	5	57.4 (42.3–72.4)	3.0 (2.1–4.6)	15.0 (5.9–39.0)
Kalongo (67), 0.5	54 (80.6)	69.8–88.8	13	24 (35.8)	25.1–47.8	1	44.8 (28.4–61.2)	2.3 (1.6–3.2)	7.4 (3.2–17.7)
Kanyege (42), 0.8	34 (81.0)	67.0–90.7	12	15 (35.7)	22.4–51.0	2	45.2 (24.1–66.4)	2.3 (1.5–3.5)	7.7 (2.6–23.7)
Kapeka 1 (27), 0.8	18 (66.7)	47.6–82.4	10	5 (18.5)	7.1–36.4	11	48.1 (21.4–74.9)	3.6 (1.6–8.3)	8.8 (2.2–38.5)
Kapeka 2 (129), 0.7	92 (71.3)	63.1–78.6	11	31 (24.0)	17.3–32.0	9	47.3 (35.8–58.8)	3.0 (2.1–4.1)	7.9 (4.4–14.3)
Kapeka 3 (31), 1.9	25 (80.6)	64.0–91.8	2	5 (16.1)	6.2–32.2	12	64.5 (42.3–86.7)	5.0 (2.2–11.4)	21.7 (5.1–99.6)
Maraam (88), 0.2	72 (81.8)	72.7–88.9	8	25 (28.4)	197–38.5	4	53.4 (39.9–66.9)	2.9 (2.0–4.1)	11.3 (5.3–24.8)
Nyabyeya 2 (14), 0.7	12 (85.7)	60.3–97.5	7	4 (28.6)	9.8–55.5	3	57.1 (20.1–94.2)	3.0 (1.3–7.1)	15.0 (1.8–176.1)
Nyakafunjo (18), 0.5	14 (77.8)	54.7–92.5	9	5 (27.8)	11.0–51.3	6	50.0 (16.2–83.3)	2.8 (1.3–6.1)	9.1 (1.6–55.1)
Rwempisi (31), 6.4	25 (80.6)	64.0–91.8	1	4 (12.9)	4.2–28.3	13	67.7 (46.3–89.2)	6.3 (2.5–15.9)	28.1 (6.1–144.3)
Rwengabi (58), 1.3	48 (82.8)	71.4–90.9	4	14 (24.1)	14.5–36.4	8	58.6 (42.2–75.0)	3.4 (2.1–5.5)	15.1 (5.6–41.7)
Rwentale 1 (89), 5.5	74 (83.1)	74.3–89.9	3	17 (19.1)	11.9–28.3	10	64.0 (51.6–76.4)	4.4 (2.8–6.7)	20.9 (9.1–48.5)
Simba (50), 0.2	42 (84.0)	70.9–92.8	5	13 (26.0)	15.3–39.4	7	58.0 (40.2–75.8)	3.2 (2.0–5.2)	14.9 (5.1–45.7)
Total (712)	568 (79.8)	76.7–82.6		181 (25.4)	22.3–28.7		54.4 (49.9–58.8)	3.1 (2.8–3.6)	11.6 (9.0–15.0)

### 3.4 Visual distribution of *Anaplasmataceae* in the study area of Uganda

**Figure 2:**
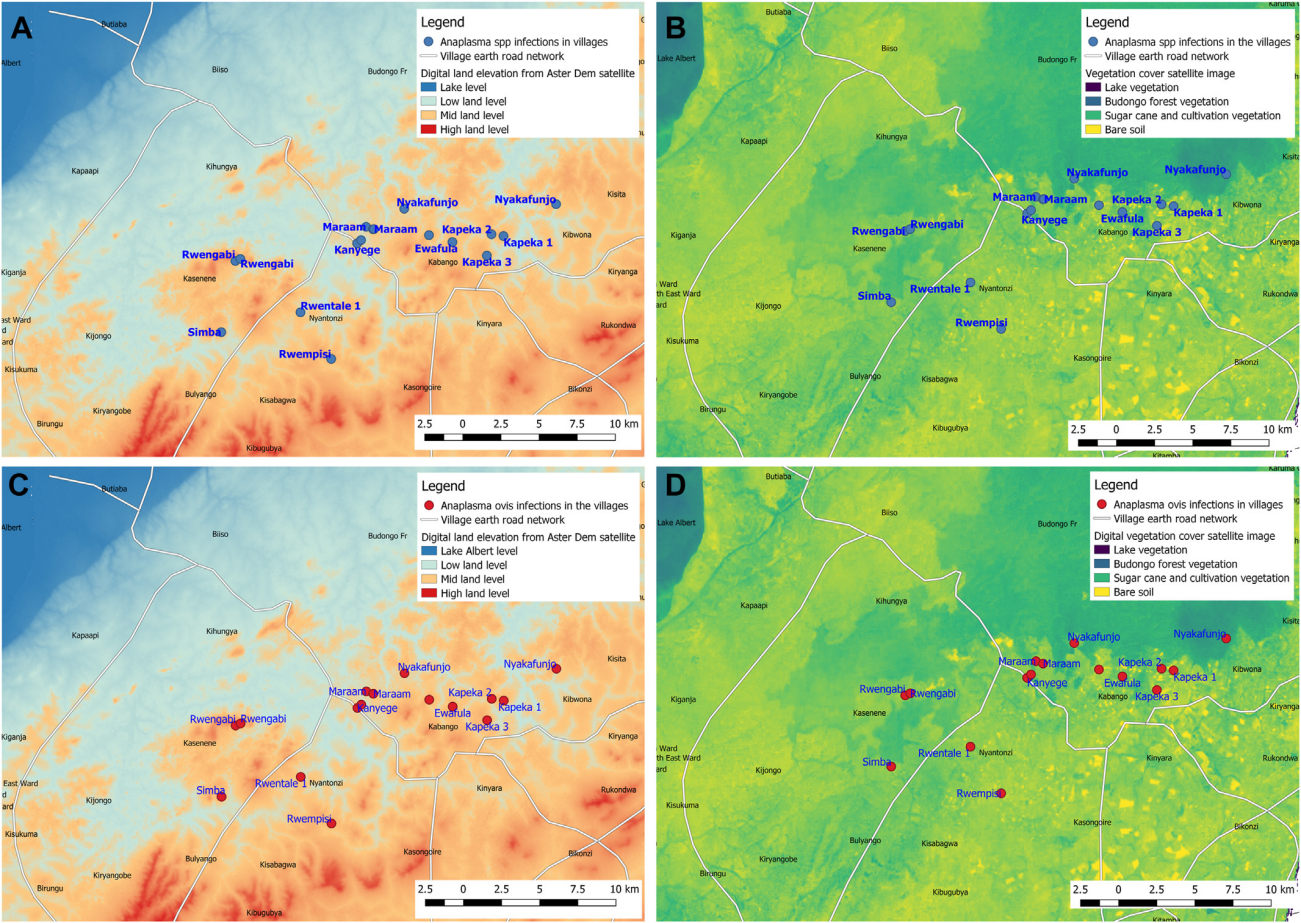
**Geographical distribution of *Anaplasmataceae* with land elevation and vegetation cover in the study area.** A = Digital land elevation showing villages with *Anaplasma* spp. B = Anaplasmataceae with vegetation cover in the villages. C = digital land elevation with *A. ovis* while D = vegetation cover with *A. ovis* in the study area.

Within the community, prevalence of *Anaplasmataceae* was in the range of 70%–86% while *A. ovis* prevalence was 12%–36% showing the importance of *Anaplasmataceae* in small ruminants in the community. Prevalence and risk of infection was greatest in Rwempisi, Kapeka 3, Ewafula, Nyabyeya 2 and Rwengabi, Rwentale 1, Kapeka 1 and all of these villages were in the midland level elevation (Figure 2A) with sparse vegetation cover (Figure 2B) as a result of human agricultural activities and commercial sugarcane production.

Villages with high *Anaplasma ovis* prevalence (Kalongo, Kanyege, Nyabyeya 2, Marram and Ewafula) were in lowland areas (Figure 2C) with relatively heavy vegetation due (Figure 2D) to their close proximity to the forest edge (less than 0.5km).

## 4 Discussion

This molecular based study has demonstrated a high-level of *Anaplasmataceae* infection in small ruminants within western Uganda. This was the first molecular study in small ruminants of Uganda and the prevalence reported in this study was much higher than reports from Kenya (40.8%) (Ringo et al., 2019) and Sudan (60.1%) (Lee et al., 2018). Here we targeted only one species of *Anaplasma* (*A. ovis*). The high levels of positivity of 16S is suggestive of other infectious agents within the small ruminant population. New strains of *Anaplasma* bacterial infections have been reported in China (Yang et al., 2016) and in Uganda (Ikwap et al., 2010), however, emphasis continues to be placed on large ruminants. Exclusion of small ruminants from policy and control initiatives will compromise community disease control efforts due to small ruminants acting as reservoirs in farming communities.

*Anaplasma* infections have previously been found higher in ovines than caprines in studies in Sudan and Kenya (El Imam et al., 2016; Lee et al., 2018; Ringo et al., 2019), in agreement with findings shown in this study. There is a need for a more holistic approach in policy for vector control in Uganda given small ruminants, especially ovines are equally infected with *Anaplasma* bacteria as large ruminants (Kasozi et al., 2019; Matovu et al., 2020; Vudriko et al., 2016), and are critical to community livelihoods. The presence of *Anaplasma* parasites in ticks at the forest edge (Punsantsogvoo et al., 2014), increased wildlife-human conflict through increased crop raiding by wildlife (Siljander et al., 2020), disrupts ecosystem health when vectors are exported from the forest to the community by wildlife during crop and animal raids and livestock grazing activities (Weny et al., 2017).Although the lack of sequencing leaves *Anaplasma ovis* identification without rigorous confirmation, we consider that our positive PCR results for *A. ovis MSP4* gene makes *A. ovis* identification the most likely interpretation. Accordingly, the risk of *A. ovis* infections in this study was found to be the same for age and sex, and this runs contrary to findings from Sudan in which risk was highest in juveniles and males (Lee et al., 2018). Infections of *A. ovis* were also found to be similar in males and females in this study, contrary to findings in Brazil in which males were found to be at higher risk (Da Silva et al., 2018). This may be due to poor animal welfare practices whereby male animals are not prioritized in disease control strategies.

This was the first study reporting the molecular epidemiology of *Anaplasmataceae* in small ruminants of Uganda, however, further studies using the 16S rRNA and other gene targets related to *Anaplasmataceae* (Ali et al., 2017; Ringo et al., 2019; Seo et al., 2018; Shi et al., 2020; Yang et al., 2016), would pave way for the characterization of *Anaplasma* infections in both small and large ruminants in Uganda to understand evolutionary changes and sources of variation for improved animal health.

There was an association between human settlement in the mid-lands and agricultural activities at the forest edge were associated and prevalence of *Anaplasmataceae*. In China, *Anaplasma* infections were associated with farming communities (Yang et al., 2016). Since ticks at the forest edge carry *Anaplasma* parasites (Punsantsogvoo et al., 2014), human-livestock activities at the forest edge increase infection rates. Increased community encroachment on the forest, such as in endemic communities of Rwempisi, Nyabeya and Kapeka 3 create an environmental interface where small ruminants are continuously exposed to disease vectors at the forest edge. Infections were found to be higher in communities closer (<0.3 km from forest edge) than those farther inland. This increase in the infection risk in the surrounding villages leads to a greater disease burden in the communities at the forest edge. The presence of zoonotic species of *A. phagocytophilum* and *A. capra* raises public health importance of *Anaplasmataceae* (Shi et al., 2020). Policy failures to prioritize animal health and devise robust policies to limit small ruminant (and human) exposure to disease vectors will continue to lead to increased livestock losses in the animal industry and impact on human health and wellbeing.

## 5 Conclusion

It is clear from the high prevalence of *Anaplasmataceae* reported in this study that anaplasmosis in small ruminants in Uganda has in general been under-reported. *Anaplasma ovis* prevalence was higher in ovines than caprines that reflects the limited community treatments on ovines during routine animal disease control activities. There is a need to include regular surveys to provide data to guide policy makers and to prioritize small ruminant health in rural communities of East Africa.

Findings in the study support that more awareness is needed to promote ecosystem health in disease control strategies to eliminate reservoirs of infections, including those that are zoonotic. Agricultural activities at the forest edge increase human-domestic-wildlife interactions (Kasozi et al., 2019), leading to increased prevalence of infection. Global health disease control strategies which promote ecosystem health and limit community encroachments on the forest edge would support one health practice. In this study, we placed emphasis on *A. ovis* due to the acquisition of suitable control DNA, however, the large number of positives for *Anaplasmataceae* suggests other species are present and this demonstrates the need to conduct additional studies in the region including targeting *A. phagocytophilium,* and DNA sequencing which were impossible to conduct in this study due to budgetary restrictions. Furthermore, differences in sample size in caprines (n = 666) and ovines (n = 46), since in this community, there were just more caprines than ovines.

## Declarations

### Author contribution statement

K. Kasozi: Conceived and designed the experiments; Performed the experiments; Analyzed and interpreted the data; Contributed reagents, materials, analysis tools or data; Wrote the paper.

S. Welburn and E. MacLeod: Conceived and designed the experiments; Analyzed and interpreted the data; Contributed reagents, materials, analysis tools or data.

D. Nalumenya, M. Namayanja, K. Matama, K. Zalwango, W. Matovu, G. Zirintunda, J. Ekou, S. Kembabazi, C. Mugasa, A. Kitibwa, D. Tayebwa, S. Musinguzi, M. Mahero, I. Ssengendo, A. Nanteza and E: Matovu: Contributed reagents, materials, analysis tools or data.

G. E.-S. Batiha and N. Marraiki: Analyzed and interpreted the data; Contributed reagents, materials, analysis tools or data.

### Funding statement

This work was supported by the Innovation Initiative Grant ID: GR003306 from the University of Edinburgh, The Commonwealth Scholarship Commission from the United Kingdom (Grant ID: UGCD-2015-168), and Busitema University Grant ID: BU/CR/156/1/Kasozi provided some of the funding related to collection and isolation of DNA from ovines and caprines.

### Data availability statement

Data associated with this study can be accessed at https://figshare.com/s/2d7b22e5a014991fae4d.

### Declaration of interests statement

The authors declare no conflict of interest.

### Additional information

No additional information is available for this paper.
